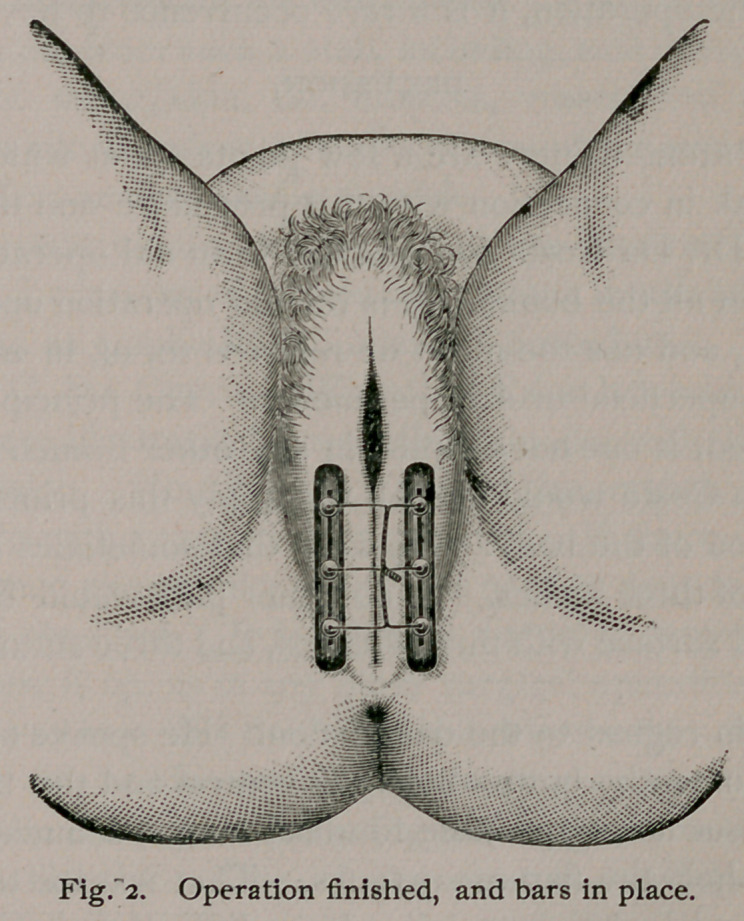# Obstetrical Society of Philadelphia

**Published:** 1889-11

**Authors:** J. M. Baldy

**Affiliations:** Secretary; 328 South 17th Street


### Society Reports.

### OBSTETRICAL SOCIETY OF PHILADELPHIA.

SEPTEMBER 5, 1889.

Dr. C. John DaCosta in the chair:

DR. JOHN DACOSTA: An Easy Method of Repairing the Perinæum.

There is probably not any operation in gynæcology which gives a woman so much relief as the proper restoration of a torn perinæum.

In describing this operation, I shall not say a word in regard to the anatomy of the perinæum, which is the same as it was a hundred years ago. The same muscles are torn now as were torn then. This subject of tear of the perinæum may seem to be a very simple matter; but when we consider that twenty per cent. of women have their perinæa torn in the first labors, and four per cent. in subsequent labors, it ceases to be a little matter, and becomes one of importance.

I do not claim anything new. The operation is the result of a combination of old ideas. It is an easy and simple method of repairing the perinæum, and answers equally well whether the tear is long or short. I thought I had something new in the use of these rubber bars, when I got it up eight years ago, but afterwards found that one of my ideas had been anticipated twenty years before.

Mr. Lane, of London, in 1860, used ivory bars with small perforations, and reports thirty consecutive cases without a failure. Dr. Thompson, of Washington, used flat rubber bars with small holes in them, and reports fifty-three consecutive cases, all cured. Dr. Thomas, after speaking of the quill suture, leads us to infer that he used perforated bars, and states that he does not recall a failure in the operation.

I do not know how many present are believers in the idea advanced four or five years ago, at the meeting of the American Gynæcological Society, "that there is no such thing as a perinæum;" but there certainly is a triangular body between the vagina on one side and the rectum on the other; and this triangular body is often torn through during labor and becomes what I call a ruptured perinæum. There are many ways of repairing it. Some are very simple, some are very striking but very useless; what I strive to do is to restore the perinæum very much as nature made it. The operation is easy, and the armamentarium is simple. We require a pair of scissors (I use a pair of blunt-pointed scissors), a perinæal needle, a little silver wire and shot, a shot compressor, and two bars shaped like the cut.

Bar, ½ size.

The operation is begun at the bottom of the tear in the vagina. With one or two fingers in the rectum, I make a little slit at the lowest point, and denude subcutaneously all the tissue that has been torn. I do not know how far up I go—it may be two inches, or even nearly the length of the finger. This depends altogether upon the extent of the tear. The important thing is to get rid of all the scar tissue. Unless this is done, good union will not be secured. After denuding up the proper distance, the scissors are turned to the right and to the left, and each side denuded. Then, with four cuts of the scissors, the loosened cicatrical tissue is removed. A denudation of this kind freshens the torn perinæum as I think no other method does. The first stitch near the bottom of the raw surface is passed three-fourths of an inch from the edge of the cut portion, buried in the tissue the whole distance, and comes out at the same distance on the other side. The needle is then threaded with silver wire and withdrawn. The second stitch is put in the same way. The third stitch is started in the skin like the others, and three-fourths of an inch from the edge of the cut, carried along just under the edge of the denudation the whole way around. This is the most important stitch of all. It was the idea of the late Albert H. Smith, when one of the physicians-in-chief at the Nurses' Home some years ago. The stitches are buried throughout, and only three are used in the operation. All that is necessary is to bring them out in nearly a straight line.

Fig. 1. Surface denuded, and stitches in place.

The wires are then slipped through slotted rubber bars on each side, and shot clamped on them. After the shot are clamped, the ends of the wires are twisted over the median line, and the ends passed through a piece of catheter. In twenty-four hours there is swelling and a certain amount of inflammation. I then cut the wires off close above the shot, and this at once relieves the tension and the pain. Any desired dressing may then be applied, if it is thought advisable to use any dressing.

What are the advantages of this operation? In the first place, you have but three stitches. I think that probably every gentleman has seen perinæ operated on where there has been deep quilting, and have seen the tissue slough out because the circulation has been so interfered with that nutrition could not be maintained. These three sutures interfere very little with the circulation, and they hold together the deep parts of the wound, which is very important. When inflammation takes place, you cut the wires over the shot, the bars spread and relieve the tension and prevent any tendency of sloughing, while still supporting the parts.

After the wound is closed, you may take a piece of cat-gut and whip up the edges in the vagina and along the line of the raphé. This is not necessary unless we want to make a very perfect job.

Fig. 2. Operation finished, and bars in place.

The operation is easily and quickly performed. I have never timed myself, and never tried to do the operation in a hurry, but I accidentally found out how long it takes.

On one occasion, in thirty minutes from the time that I began, I had operated on two cases, and this included the time necessary to put one patient under ether from perfect consciousness to unconsciousness. The denudation is accomplished in four or five minutes.

This is a different operation from that in which the denudation is made in curved lines, and where another operation is required for any existing rectocele. The operation described above will include also a rectocele. It is better than another popular operation, which does not restore the triangle which nature made, but makes a beautiful skin-flap, which looks well from the outside, but affords no support.

I do not claim anything novel. It is simply a combination of ideas that I have picked up from time to time. In regard to the results of the operation, it is a rare occurrence to have a failure.

DISCUSSION.

DR. J. PRICE.—There are a few points about which I should like to speak in connection with this procedure and like procedures. As Dr. Da Costa has said this is an old operation, and is illustrated in all the books. It is the old operation upon the posterior wall, and has the merit he referred to, of, in many cases, making a superficial or skin perinæum. The principle of suturing described, is one not adopted in any other branch of surgery, and Dr. Da Costa would himself not apply this principle in any other portion of the body. He says that sometimes he denudes a distance of three inches. In no other part would he approximate such a surface with three sutures, and three sutures will not close it.

A word in regard to the denudation. He speaks of four clips of the scissors—the button-hole, the central and the two lateral. In many cases it is impossible to make such a denudation. You will button-hole the flap many times. That was the trouble with the Smith and Jenks operation. It is difficult to make a clean denudation in the midst of scar tissue by such a method.

One of these illustrations shows what takes place in many perineal tears. The skin-perinæum side is not harmed; but if you place your finger in the suclus on one side, you will find a sense of resistance which is absent on the other side. The sulcus is a deep one, and is a lateral tear. As has been remarked by Dr. Deaver: "It is for all the world like the lateral cut for stone." In such a case the procedure is almost a unilateral one to bring up the pelvic floor. It is just such a state of affairs that Emmet had in view in his classical operation for the restoration of the pelvic floor or diaphragm, and he has most beautifully succeeded.

In regard to the use of this needle. Dr. Da Costa has referred to the fifty-three cases reported by Dr. Thompson of the Columbia Hospital; but he lost one or two from tetanus, and this bayonet was at the bottom of the tetanus. I look upon this needle as wholly unjustifiable in any surgery. No man has a right to have such a thing among his instruments. I am surprised that more do not die from such a stab, including, as it does, incongruous masses of tissue, skin, fat, muscles, vessels and nerves. I remember, while a student, of seeing a death from such a stab. I use the smallest sewing needle possible.

These procedures are very old, and are illustrated in all the old works. I consider all two-or-three-stitch methods of closing the perinæum as emphatically imperfect procedures.

DR. JOHN C. DA COSTA.—What Dr. Price has said in regard to one of these illustrations has nothing to do with the subject under discussion. He refers to a tear of the vagina, which has nothing to do with a tear of the perinæum. If there is a line of cicatricial tissue on one side, we do not need to denude both sides to repair the condition. It is a simple matter to remove the scar tissue and sew it up, as in any other surgical operation.

I am sorry to hear this tirade against this needle. Some very able men use this needle, and they get very good results. Albert H. Smith, who did a good deal of gynæcological work, used a needle much like this. One of the most successful abdominal surgeons in Philadelphia uses a needle much like this. Surgeons in all branches of surgery use needles very like this—either a little more or a little less curved. One who came from Europe a year ago showed me a long, curved needle which he brought with him and said was Tait's needle. It was precisely similar to one which I have had in my box for some years for use in complete laceration of the perinæum. This is only the Baker-Brown needle modified.

I do not know that Dr. Price has said anything against this operation. He has talked a good deal about the needle and about a tear that does not apply at all. I can only say that, despite his fears, the operations are almost uniformly successful. Any one who can do the ordinary quill operation can do this. After analyzing the various operations eight years ago, I found that the best results were obtained by the old-fashioned operation. The quill operation, however, made a V-shaped sinus to the bottom of the wound, and sometimes caused a great deal of trouble; and it was to overcome this objection that I substituted the hard rubber bars with the wires running through.

Dr. JOSEPH HOFFMAN: A Report of a Series of Abdominal Sections, with Special Reference to Complications.

In bringing this report before the society, my object is not to apologize for results, nor to explain failures, except so far as they may be made to offer escape from discovered error in operation. The list includes a series of thirty-three operations, consecutively performed. None are omitted. Selected cases are well enough for any *eclat* they hope to gather about them, but they teach nothing and record nothing but the good fortune of the operator, and leave nothing for comparison, whereby his work may be truly estimated. I believe that selected cases are commonly selfish advertisements. A report of a number of hysterectomeis, all successful, saying nothing about nearly an equal number that have been failures, can be only misleading. This is true of all other operations where failure may teach us as much, nay more, than success.

The list of operations may be classified as follows: one strangulated ventral hernia; one appendicitis and hæmatocele; sixteen cases of ovarian and tubal disease, with adhesions, inflammation, and occlusion of the tubes, with one death, the result of sepsis. Where the infection came from, for a long time puzzled me. Months after the death I learned that the patient had had a miscarriage brought on instrumentally, and the mystery was solved. In other words, I believe the tubes were septic, and gave rise to the peritonitis.

By this case I believe there is sufficient learned to warrant the practice of cauterizing the tubes after ligation and section in all doubtful cases where there is the least suspicion of infection. If this is not done, they should be thoroughly disinfected and the abdomen drenched. I have had no other death from like cause, or from peritonitis from any cause. Six cases of pyosalpinx, one occurred during pregnancy, and the other was done to save life. The woman miscarried the fourth day after the operation but made an excellent recovery, though her pains were very great during miscarriage, and were only controlled by the free use of morphia and atrophia. All cases of pyosalpinx recovered. They are all working in comfort save one, who has lately died of tuberculosis. The last case was strongly in support of Bernutz's view, that pus in the tubes is a forerunner of general tuberculosis.

In two cases the gonorrhœal origin of pyosalpinx is well established; in two the history points to post-puerperal origin; in the remaining two the origin is doubtful, though in one of the cases I strongly suspect a specific start.

One died of shock—never coming out of anæsthetic. She was a hard drinker.

In two cases the tumor removed was dermoid. Both were small. In one of these cases the uterus was rudimentary, though the woman had for a long time worn a pessary for a so-called displacement, introduced by a specialist in gynæcology. There is sufficient commentary here on the use and abuse of pessaries without further remark. In two cases exploratory incision was made. In both the women recovered quickly. One of these soon after died after tapping; from what cause I do not know. I visited her for a day or two after tapping her, and was told that the patient was feeling so well that no further visit was necessary. In a week, or thereabouts, I learned of her death in the hands of another. The whole air of the matter was unsavory, and I am not sorry to remain in ignorance concerning it. The second exploratory incision was due to an error in diagnosis. The uterus was retroverted, a miscarriage having occurred a short time previously. There was a peculiar thickening of the right broad ligament, which immediately led to the blunder. I examined the patient on my table soon after her recovery, and had

I not known that I had erred before, the condition was such that I would have done so again. Two small ovarian cysts; both recovered. One case of omental hernia, one case of extra-uterine pregnancy. One case of operations for adhesions due to previous operation. The result has now a greater measure of success than I hoped for a short time ago.

The drainage tube was used in fourteen cases. I believe I would have had a better chance of saving one of my deaths had I used it. I have never had a death from its introduction. I have had but one fistula persisting after its use, and this now gives every sign of closing. In only one case has there been a discharge of the ligature. The patients operated upon are now all living but four. They are all able to do their work comfortably save two. One case, I believe, is reported to have had another operation. She was a most ungrateful baggage, and I trust she will tarry a long while on earth for the experience she will bring to others. I have had one case of hernia after simple section. The woman was fat, and neglected her bandage. In two cases where it existed previously to operation it still is present. I did not really operate for its cure. I have found drainage and flushing the abdomen to be of the greatest service in cleansing the abdomen of *débris*, and believe them indispensable. Free saline purgation, or, when the salts are not retained, mercurial purgation, is of the greatest benefit in severe wind-pains. These are probably more frequently the cause of pain soon after the operation than inflammation, though there is no doubt that here, also, these purges are of undoubted value. In the question of diagnosis, I find it is much easier to say there is a lesion than to map it out exactly or to define it. I have found marked trouble in cases where expert examination pronounced disease absent; in others, where one thing seemed to be the trouble, another was found present. So far as pain is concerned, it does not always indicate the spot of the lesion. I have found one side the most diseased when it was freest from pain.

In the thirty-three operations recorded, two deaths have occurred. No patient was operated on by myself more than once. The first death occurred early in the series. In the last twentysix cases there has been but one death. The last eighteen cases have been without death.

DR. J. PRICE.—The mortality of this group of cases is exceptionally low. I had the opportunity of seeing most of them and counselling the procedure. Dr. Hoffman states that in some of his cases the lesions were not very marked. To me they were decidedly marked. The patients call one's attention to the seat of pain. Some of them can ride in street-cars, but experience pain at the crossings. Others cannot ride in the street-cars on account of the pain. Not over an hour ago a patient left my office and walked home because she suffered too much pain to ride. It is important that we should do this work publicly, and make public demonstrations of the angry nature of these troubles. Many of these patients have been married from one to fifteen years without conception. Some have conceived; some have had one child, but none have had more than one.

The pus cases and the extra-uterine cases interest me most. Three weeks ago I paid for carriages to send two cases to the hospital. I sent them there at five or six o'clock in the afternoon and operated at nine o'clock the next morning. On the same day I saw, in consultation, three cases, in which I urged immediate operation. This was not done, and they all died; my two cases recovered. It is curious how slow men are. They do not understand the importance of promptitude. Some weeks ago I urged operation in a case of disease of the appendix. The operation was done promptly, and the patient lives; she had suffered ten previous attacks. Surely that life was saved. Treves recently gives a similar case, and Dr. Baldy has had another. In the Pittsburgh *Medical Journal*, there were recently reported three or four cases of death by a man who says that the appendix is not in the abdomen.

A word in regard to flushing the peritoneal cavity. The gravity irrigator is a beautiful machine. It floats up everything, and you have less manipulation and traumatism. The drainage is surer, and you remove the tube sooner. There is less shock, and the reaction is rapid. There is less rapidity of pulse and elevation of temperature.

DR. J. M. BALDY.—I wish to emphasize the point so prominently brought forward by Dr. Price,—the importance of promptness in these cases, especially the imflammatory diseases. The case of extra-uterine pregnancy which I had a few weeks ago brought this to my mind more forcibly than it had been for some time. This was a case in which I little suspected extra-uterine pregnancy. My diagnosis was pus-tube. I operated and found extra-uterine pregnancy. It was evident that the woman could have lived only a few days if the operation had not been performed. There are many such cases occurring in this city. In many the operation has been advised but not performed. The probability is that many of these cases die promptly. I know of a number of cases similar to those reported by Dr. Price. These cases are not rare, and besides ectoptic gestation there are other things that will kill quickly. A blow or a kick may rupture an abscess and in a few hours the woman will be in the coroner's hands.

With regard to the slight lesions referred to by Dr. Hoffman, I had such a case to-day,—that of a young woman married three years. She had one child in the first year of marriage. She now comes, stating that she has been a continuous sufferer ever since. She arose from bed a few days after her confinement. She then "took cold" and had to go to bed for two months. The case turned out to be one of ovarian cyst. She begged that, if possible, one ovary be left and that she be not made sterile. After removing the cyst, which was somewhat of a surprise, I passed my fingers to the other side and found the appendages apparently normal, but slightly adherent. I left them, but I now think that I made a mistake. Examining the tube of the side removed, I found the fimbriated extremity entirely occluded and the tube utterly useless. The other tube is probably in the same condition, but I did not bring it to the incision to examine it, trusting entirely to my fingers to tell the tale, and they only detected "*slight disease.*" Many of the cases spoken of as cases of slight lesions are just such cases, and are as bad as the worst pus cases, so far as child-bearing or the comfort of the woman is concerned. They should every one be removed without hesitation.

DR. JOHN HOFFMAN.—The gentleman, I think, misunderstood my remarks so far as the slight lesions were concerned. I simply mean that some of them were not easily discovered.

I had intended to discuss somewhat the complications of the work, and hoped that they would be referred to in the discussion. Without going into these complications, there is one sequel that I have noticed, and I have noticed it after careful work with which I can find no fault; that is, subsequent hemorrhage. This frequently takes place three or four months after the section. I have had a case go six months and then menstruation recur, continue a few months, and be followed by an attack of flooding. If there is any explanation of these cases I should like to hear it. In one case where the congestion was very great, and where I curetted the womb, there was a post-operative hæmatocele. In some cases, where there is no discoverable lesion, these hemorrhages will recur monthly, or bi-monthly and are a source of discomfort.

I wish to call attention to a complication that occurred in my case of extra-uterine pregnancy. The woman did excellently until the tenth day. In this case the bowel had been torn through, but was sutured. The abdomen was packed with a yard of lint and the hemorrhage controlled. Secondary hemorrhage occurred at the end of ten days; it was so abundant that it soaked the bed-clothing. I raised the foot of the bed and kept her quiet for two or three days and the hemorrhage ceased. The drainage tract healed up entirely and there is no sign of fistula.

DR. J. PRICE.—This subject of hemorrhage following removal of the appendages is one about which I know very little. I have been puzzled to explain it. I am satisfied that I have included the nerves about which so much has been said by Johnstone and others, but which have been known to exist for two hundred and fifty years. I have tied the tube close to the uterus, and in some cases curetted the remaining portion, and have even used the cautery. Still, in some of these cases, at the end of one, three or six months, hemorrhage has occurred. I have looked upon this as rather of a safety-valve action. I am rather pleased to see them bleed. I think that then the establishment of the menopause is more satisfactory. Those cases which have not bled have complained more of creeps, flushes and those uncomfortable phenomena incident to the menopause. I have never seen the bleeding as irregular or profuse as before the operation. It is rather curious that this is more common in the pus cases done to save life than in the fibromas where the operation is done to establish the menopause. Bleeding occurs in probably ten per cent. of the fibroid cases, and in twenty-five per cent. of the pus cases. These figures are, however, only approximate. My experience is, that the bleeding never continues more than a year.

So far as I am aware, operators have not put on record a single case of supernumerary ovary. I have never found a supernumerary ovary. When I have finished I am satisfied that they do not exist.

DR. J. M. BALDY.—So far as my experience goes, I cannot say that any one class of cases is more liable than another to suffer from this hemorrhage. I used to promise that the menses would cease, but I have had bitter experience, and now makes no promises on that score. I do not believe much in the "safety-valve" theory. I do not think that it is a good thing for them to go on bleeding. In one of the cases in which Dr. Hoffman removed the appendages cleanly, there was monthly bleeding. I afterwards saw her uterus curetted, and there was undoubtedly malignant disease. I do not mean to say that in a large number of cases there is malignant disease, but in this one case there was.

I have never found any other thing that satisfied me in regard to the cause, with the exception of habit. The woman has been in the habit of menstruating for years, and does not get over it immediately.

Supernumerary ovaries I have never seen, and I believe all the talk we hear about them is nonsense.

DR. JOHN B. DEAVER.—I have seen many cases of the kind described. I do not see the philosophy of the good effect to be derived from the continuance of the bleeding. I am inclined to think to the contrary. I have seen it follow occluded tubes with displaced and adherent ovaries. I have seen it last for a long time, and not yield to internal remedies.

In regard to the question of supernumerary ovary, I have not looked this up in the dissecting-room, but at present I cannot recall one instance of supernumerary ovary.

DR. WM. E. ASHTON.—The point in regard to supernumerary ovaries is referred to by Winckle, and I think that he states that sixty-seven per cent. of women have supernumerary ovaries.

DR. JOSEPH HOFFMAN.—I have succeeded in benefiting these hemorrhages by the use of the curette. It seems to me that in many cases the hemorrhage is due to hyperplasia of the intra-uterine mucous membrane. In one case the bleeding comes regularly every four weeks, and the woman feels better when it comes. In other cases, I have known women to bleed so much that they were frightened. After the bleeding had continued a week or ten days, it stopped. In one such case, I used the curette, and the woman is now well. I do not believe that one explanation will cover all of the cases.

The case mentioned by Dr. Baldy is the one to which I referred as having been operated on again. The woman had Bright's disease. An extensive bleeding occurred when the kidneys were doing the least work, secreting not more than an ounce of albuminous urine in twenty-four hours. I do not believe that she had malignant disease. I should want a microscopic examination to satisfy me. The patient was red-faced and hearty and felt well, with the exception that she bled.

Dr. JOSEPH PRICE: Two Operations for Extra-Uterine Pregnancy.

The first case I have to report is that of a white woman, aged 35, nursing a child of thirteen months. Menses appeared on the fourth month of lactation, and remained perfectly regular at intervals of twenty-seven days; four days duration; were absent two periods, followed by paroxysms of pain and collapse. At this point I saw her, and operated immediately for ruptured tubal pregnancy. I found about a quart of clotted blood in the peritoneal cavity; tubal rupture left side; hydro-salpinx right side; clean removal of both sides; irrigation; drainage; recovery.

The second case, occurring in a pure negress, is of great interest. I am not satisfied, from the microscopical appearance, that it is a true *ovarian* pregnancy. Ovarian cysts are very rare in true Africans. In the blood cyst I found something for all the world like placenta and membrane. I do not wish to put this on record as an ovarian pregnancy until I receive the report of Dr. Henry Formad, the pathologist. There also existed in this case a hydro-salpinx of the other side,—both demonstrating most beautifully the causal relation of tubal disease to ectopic gestation. One point of great interest, in connection with these cases that survive the rupture and go into the hands of the surgeon, is the marked difference in the character of the hemorrhage from those that go into the hands of the coroner,—and they are numerous. In the latter cases, the hemorrhage is overwhelming, and the abdomen is found full of blood. The surgeon finds probably one-fourth the blood. Dr. Formad, the coroner's physician, tells me that in one case he found the peritonæum deluged with blood, and the little fœtus sitting, or washed up, on the pancreas. Its object was probably to try and escape a possibility of electrical treatment.

Dr. J. M. BALDY.—As Dr. Price states that ectopic gestation is rare in the full-blooded negro, I should like to place on record, by the side of his, such a case. The woman had been having children at regular intervals of two years for eight or ten years. The last time, when her time came to become pregnant, it proved to be pregnancy of the tube. She was operated on and saved.

Dr. JOSEPH HOFFMAN.—In extra-uterine pregnancy, the woman has usually been sterile for a long time ; but in my case the patient had a child fifteen months old,—had missed four weeks, and thought she had caught cold. At the fifth week she had a period. The bleeding continued, and an examination showed a mass in the pelvis. I did not attempt to diagnose its exact nature. I had no idea that it was an extra-uterine pregnancy until the abdomen was opened. If I had been thinking of it, the diagnosis could easily have been made.

Dr. M. PRICE.—There is one point to which I would call attention ; and that is, that in nearly every case falling into the coroner's hands the fœtus has been found. This is simply because death has occurred so rapidly that the fœtus has not had a chance to disappear. In the cases coming to the surgeon, there is not so much bleeding, and the delay gives the fœtus a chance to be digested by the peritonæum. I doubt whether fœtuses, such as have been found by Dr. Formad, could have remained a few day without absorption. Many cases have been considered doubtful because the fœtus has not been found; but the fœtus has been digested, and all that remains is the placenta, membrane and ruptured tube.

Dr. J. M. BALDY reported a case of *Fibro-Cystic Tumor of the Uterus.* Mrs. A., age 35 years, married, no children. Has had a lump in her abdomen for fifteen years, which remained quiescent until within the last two years, since which time it has grown rapidly. Menses have gradually become irregular and profuse; bowel and bladder symptoms have become severe; pus has appeared in the urine; abdomen is constantly swollen, and very painful; general health has begun to suffer severely. Examination showed a uterine tumor, and its removal was advised, the dangers of the operation being fully explained. Operation was eagerly accepted. The tumor was removed one week ago last Tuesday, and proved to be an extremely nodular fibroid, which had undergone cystic degeneration in part, and in other parts is quite œdematous, as can be seen by the specimen which is here before you. The mass was firmly wedged into the pelvis and was delivered with the greatest difficulty, leaving practically no pedicle at all. The case was treated by supra-vaginal amputation, a wire nœud being first placed around the lower portion. The stump was treated by the extra-peritoneal method, as advocated by Bantock. After the tumor had been cut away, there was left a stump with a diameter of over three inches; this was gradually trimmed away until it was reduced to about an inch and a half in diameter. The operation was altogether the most trying and most difficult one of this kind I have ever performed or seen.

This case presents the opportunity for a few remarks on the method of treating the pedicle in hysterectomy, and on the use of electricity in fibroid tumor of the uterus.

There are two methods of treating the stump,—the intra-peritoneal, as advocated by Martin, and the extra-peritoneal, as advocated by Bantock. All other methods devised or proposed are simply modifications of these two, and are far from being as good as the originals. A so-called half-way method proposed by Kelly last winter has so many objections for general application, that it is hardly worthy of consideration, excepting for picked cases; and these must be cases of the simplest kind, with a pedicle which can be easily dealt with. In the "*New York Medical Journal*," for July, Douglas has called particular attention to the defects of this departure.

What we want are results, and in questioning different gentlemen who are experimenting with the so-called improvements in hysterectomy, I find almost universally that their losses amount to from thirty per cent. to fifty per cent. The patients who get well may do so quickly, and the operations may be very beautiful theoretically, but the results are murderous! Until a larger number of cases have been reported, and the results are very decidedly better, I prefer to pin my faith to one or the other of the two original methods. Of these two, the results obtained by the extra-peritoneal method are, at present, very decidedly the best, and have proven eminently satisfactory in my hands. Martin, by the intra-peritoneal method, reports a series of eighty-four cases, with twenty-five deaths. Later, he has thirty cases, with three deaths; and still later, he has "another series with good results;" and last, "a series with bad results." And so, after an experience of much over one hundred and twenty-five cases, he ends up with a series so bad that he does not publish it. In contrast with this stands Bantock's record, by the extra-peritoneal method, of fifty-seven cases with only twelve deaths, and his results continually getting better to the end. He now has a run of thirty or forty cases without a death. These figures speak for themselves. After all his experience, Martin ends by saying, "so I think we must wait for a larger number of ca ses before deciding this question."

Of the twenty-five deaths met with by Martin in his first eighty-four cases, fifteen died of "bleeding, embolism, and collapse," all of which, of course, mean hemorrhage. Now by the extra-peritoneal method, these would have all been saved, as bleeding cannot possibly occur if the wire does not slip or break. Again, ten of Martin's cases died of sepsis; this also is much less likely to happen by the extra-peritoneal method, as all cut surfaces are outside the peritoneal cavity, in plain sight and under perfect control.

When Martin has finished his operation and drops the stump, as I have seen him do, the appearance to the naked eye is simply perfection, and one carries away the feeling that everything is cleaner than the stump of an ovariotomy. On the other hand, when the stump is left outside, as I was taught by Bantock, the after treatment is often tedious, and the convalescence prolonged. If the stump is not perfectly dry it is apt to suppurate and, at best, it is an unsightly affair. But when we contrast the results, there can be but one choice, if we give proper consideration to the safety of our patients. Not only are Martin's own results bad, but in the hands of less expert and experienced operators the mortality is very high. Even Bantock has lost four out of five cases by the intra-peritoneal method. By the extra-peritoneal method, five or six of us here in Philadelphia can now put on record twenty or more cases with only about two deaths in the lot, and those were cases in which there was extensive cancerous involvement of vital organs. In fact, our mortality is about as good as that in ovariotomy.

The use of electricity in fibroids in not without its dangers and impossibilities. Such a case as that before you is wholly beyond the reach of this palliative agent. To have done any good to that tumor, it would have to be punctured, and this large cyst which you see emptied. To have done so in this case would have required a puncture four or five inches deep; the needle would have to have penetrated the whole length of the tumor. Dr. Massey punctured one of these cystic tumors (if I recollect correctly) last winter, and the patient very promptly died of sepsis. At the June meeting of this Society, Dr. Price presented two specimens of fibroid tumors. One was a large œdematous myoma, containing blood-vessels as large as the iliacs, and as he then said, one might as well have tried to dissipate the iliacs themselves as those vessels ; a puncture of any one of them would have meant tremendous hemorrhage. The second specimen was a fibro-cystic tumor with nothing but a thin membrane between the cyst cavity and the uterine cavity, the membrane being lined with a mass of blood—sinuses as large as one's little-finger. An attempt to puncture that case would have meant almost instant death. And so it is with many other specimens on record. The fact is plain that there is a large class of fibroid tumors totally unfit for the electrical puncture ; and to make the danger in these all the greater, they cannot always be differentiated. For instance, the fibro-cystic character of the specimen before you was not even suspected during life, although repeated examinations were made. There is plenty of material here for earnest thought, and it ought to be a warning against blindly rushing into the use of electricity in all cases, simply because the enthusiastic advocates of this treatment fail to bring out its dangers, and, in fact, only too universally hide them.

DR. J. PRICE.—This specimen is very interesting, and the result very happy. Notwithstanding this is one of three cases in the hands of a beginning operator in hysterectomy, it is one of three successes. This is a rare specimen. It is found only five times by Atlee in three hundred and seventy-eight ovariotomies, and then as a result of mistaken diagnosis. This is clearly a case of cystiform degeneration of a fibroid. It is particularly interesting as occurring in a colored woman. I know of only two cases in colored women.

It is sometimes imagined that the surgeon submits all these cases to operation, but many of them are advised to go home, and everything is done to relieve their discomfort until the menopause. Much progress has been made in the last few years in perfecting this operation, and the progress has been chiefly in the treatment of the pedicle, and all successful operators now regard the extra-peritoneal fixation method as the most important step. In ovariotomy, the treatment of the pedicle began right.—Mc-

Dowell dropped the pedicle. Nathan Smith and John Light Atlee did the same, and the patients recovered. Spencer Wells fell into error in treating the pedicle externally, and it was thirty years before the vantage-ground was recovered. A comparison of the results given by Dr. Baldy, with the tables of Dr. Martin and of Dr. Bantock, would be interesting. I think that he errs in regard to Bantock's cases. I think that Bantock had seventy-two cases with twelve deaths. He rejects fifteen cases—two done by enucleation, and thirteen he throws out for some specific reason. Tait has had thirty-two cases without a death. Keith had thirty-eight or forty, with three deaths. All of these were done by the extra-peritoneal fixation method.

Some of the recent attempts to claim originality, and devise something new by radiating sutures and the old-fashioned churning method of pumping the stump up and down, is foreign to all surgical principles of rest, fixation and immobility. By such methods the risk of hemorrhage and sepsis is very great. The extra-peritoneal method is to me quite an ideal method. While, as Keith has said, the flat clamp is a clumsy and unsurgical instrument, and makes it almost impossible to close the wound nicely, the *Koeberle noeud* overcomes these objections, and brings the stump into the lower angle of the wound. There is little exposure of the abdominal cavity, and sepsis is very rare. Considering the risks of the method of dropping the stump, I think that the extra-peritoneal fixation method is much superior. You cannot discuss the subject by using the history of ovariotomy as an argument. The conditions are different and the tissues are different. I have screwed up the *noeud* as often as six times before the stump was perfectly dry, and reduced it from the thickness of my wrist to that of my finger. In Philadelphia, the results of extra-peritoneal, supra-vaginal hysterectomy are better than those of ovariotomy. We have a stump in the abdomen after ovariotomy; after hysterectomy we have nothing.

DR. M. PRICE.—I was present at this operation, and it was one of the most difficult hysterectomies that I have seen. The tumor was pelvis-bound and the whole cervix was diseased and thickened by this enlargement of fibrous tissue. The result speaks well for the extra-peritoneal method.

DR. JOSEPH HOFFMAN.—It seems to me that the term "ideal" applied to the intra-peritoneal method is a misnomer. The term "ideal" should be applied to that operation that gives the best results, and not to the one that looks the prettiest. Dr. Price has given us the key-note when he states that the conditions met with in tying off an ovarian tumor and tying off the stump of a hysterectomy are not the same.

The only thing that can be claimed by the modifications that have been introduced is, that they obviate the sloughing. But if the stump is treated by the dry method, there is absolutely no sloughing. Another advantage claimed for the intra-peritoneal method is that the incision heals more readily. I do not believe that this is proven; I do not believe that the incision is closed when the patient is pronounced cured.

DR. J. C. DA COSTA.—I think that the deaths from hysterectomy in Philadelphia have been more numerous than are imagined. I know of deaths that have not been reported. Thomas, in his book in regard to the removal of intra-mural fibroid tumors, says that the favorable cases have a wonderful faculty of getting into print which does not characterize the unfavorable cases. The same remark would well apply to hysterectomy.

Dr. M. PRICE.—I have no doubt that Dr. Da Costa is right. I know of two cases where the belly was opened by operators who did not know what they were going in for. In one case there was a solid tumor weighing fifteen pounds, and the man thought that it was an uncomplicated case of ovarian tumor. Such cases should not go on record alongside of cases done by expert operators.

Dr. J. C. DA COSTA.—The cases to which I referred did not belong to the class spoken of by Dr. Price. I had in mind one gentleman who stands high as a surgeon. He knew perfectly well what he intended to do.

Dr. JOSEPH HOFFMAN.—A fairer method would be a comparison of the methods of the best men. Keith speaks of the great difficulty of getting a stump. Now, if a man does not find a stump, he makes one. The sinking of the clamp is not met with now as often as it used to be.

Dr. J. M. BALDY.—When I said that, in the experience of a half a dozen men in Philadelphia, there were twenty cases with but two deaths, I did not attempt to include all cases operated on by every one. I have known of a surgeon, who, during the operation, took his dirty eye-glasses off his nose, and demonstrated by touching with them the different parts of the tumor to those around, and the patient died. I do not include such cases. Such men do not report their cases. I refer to the men who report *all* their cases, whether good or bad. We can only argue from the results of such men.

In regard to sloughing of the stump, I have been unfortunate. I have had a good deal of sloughing, because I have not seen that the stump was dry. In this case that was impossible. The sloughing is all outside, and is under perfect control. I have not seen the pulse go above 100°, or the temperature above 99° or 99.5°, after the operation. Talking at Newport with men who had modified the operation in various ways, I found that they were losing about three out of six cases.

I do not by any means operate on all cases of fibroid. I have now operated three times, all being successful. In the first two cases the operation was urgently demanded, and in the third I was forced to do hysterectomy. I started to remove the appendages, but there was so much hemorrhage that I thought it safer to remove the uterus. During the past summer, I have sent home four cases of fibroid, and advised that nothing be done. The vast majority of cases can be carried along until the menopause, or until they die of something else.

J. M. BALDY, M. D., Secretary.

328 South 17th Street.

## Figures and Tables

**Figure f1:**
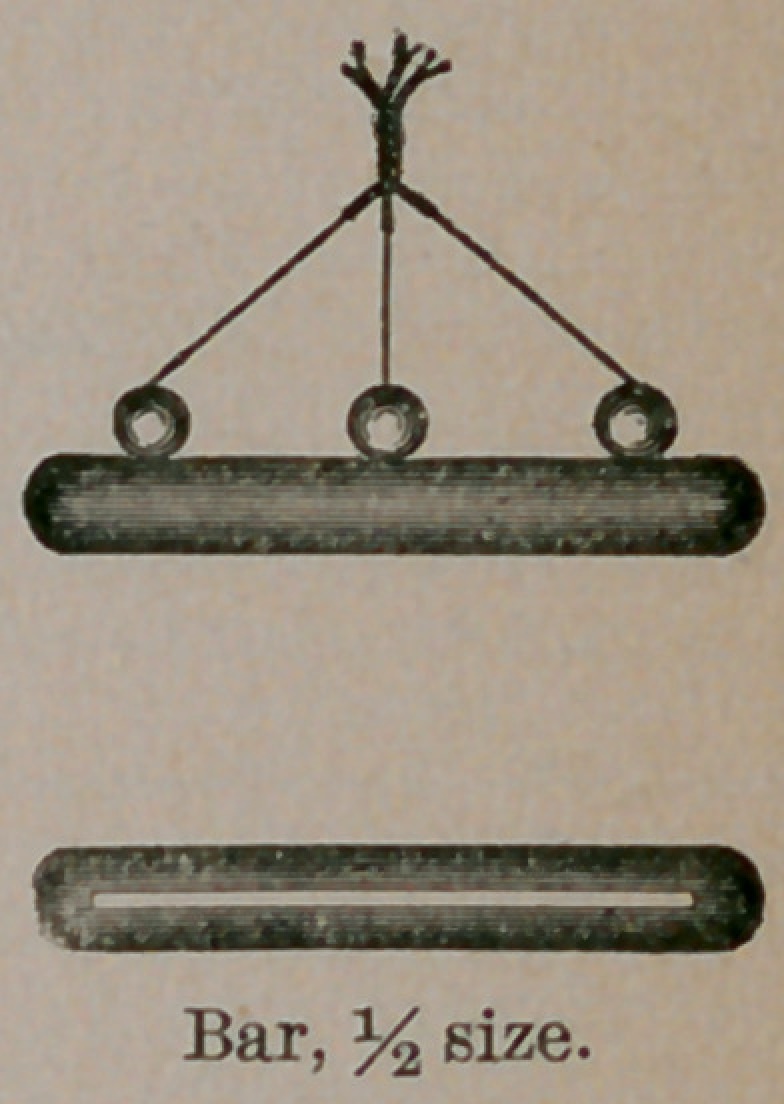


**Fig. 1. f2:**
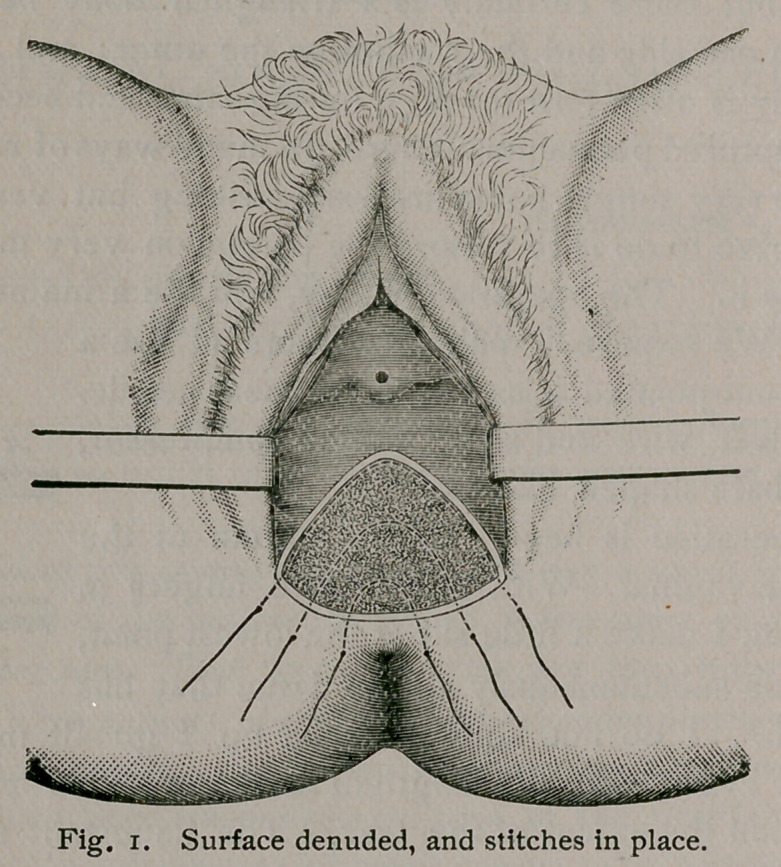


**Fig. 2. f3:**